# Evaluation of Bone Metabolism in Critically Ill Patients Using CTx and PINP

**DOI:** 10.1155/2016/1951707

**Published:** 2016-11-29

**Authors:** Alexandra Gavala, Konstantinos Makris, Anna Korompeli, Pavlos Myrianthefs

**Affiliations:** ^1^School of Health Sciences, Department of Nursing, “Agioi Anargyroi” General Hospital, National and Kapodistrian University of Athens, Noufaron & Timiou Stavrou, 14564 Kaliftaki, Nea Kifissia, Athens, Greece; ^2^Clinical Biochemistry Department, KAT General Hospital, Kifissia, Athens, Greece

## Abstract

*Background*. Prolonged immobilization, nutritional and vitamin D deficiency, and specific drug administration may lead to significant bone resorption.* Methods and Patients*. We prospectively evaluated critically ill patients admitted to the ICU for at least 10 days. Demographics, APACHE II, SOFA scores, length of stay (LOS), and drug administration were recorded. Blood collections were performed at baseline and on a weekly basis for five consecutive weeks. Serum levels of PINP, *β*-CTx, iPTH, and 25(OH)vitamin D were measured at each time-point.* Results*. We enrolled 28 patients of mean age 67.4 ± 2.3 years, mean APACHE II 22.2 ± 0.9, SOFA 10.1 ± 0.6, and LOS 31.6 ± 5.7 days. Nineteen patients were receiving low molecular weight heparin, 17 nor-epinephrine and low dose hydrocortisone, 18 transfusions, and 3 phenytoin. 25(OH)vitamin D serum levels were very low in all patients at all time-points; iPTH serum levels were increased at baseline tending to normalize on 5th week; *β*-CTx serum levels were significantly increased compared to baseline on 2nd week (peak values), whereas PINP levels were increased significantly after the 4th week.* Conclusions*. Our data show that critically ill patients had a pattern of hypovitaminosis D, increased iPTH, hypocalcaemia, and BTMs compatible with altered bone metabolism.

## 1. Introduction

Critical illness may accelerate or lead to altered bone turnover due to prolonged immobilization, nutritional defects, inflammation, vitamin D deficiency, exposure to medications that affect bone and calcium metabolism, and endocrine dysfunction. These factors, separately or in combination, may contribute to increased bone resorption and decreased bone formation [1]. This accelerated bone turnover may contribute to the burden of morbidity and mortality observed in survivors of intensive care [2, 3]. In patients with prolonged critical illness, circulating biomarkers of bone resorption are substantially elevated whereas markers of bone formation are low [4]. Also, the measurement of bone turnover markers (BTMs) reveals markedly enhanced osteoclastic bone resorption that is uncoupled from osteoblastic bone formation [5].

Such an imbalance of BTMs may predispose critically ill patients to impaired fracture healing, osteoporosis, and increased risk of new fractures during intensive care unit (ICU) stay or rehabilitation. A recent retrospective case-cohort study revealed a significant increase in fracture risk in survivors of critical illness, implying a clinically relevant impact of the reported alterations in bone biomarkers [6].

Although data are limited until now a positive association (moderate evidence) between critical illness requiring intensive care admission and increased bone turnover exists according to a recent systematic review [1]. However, the extent, risk factors, and the nature of the relationship between critical illness and bone turnover are not yet clearly understood [1].

Also, studies have employed different methodologies and recruited heterogenous populations (differences in ages, gender, and population sizes) with varied duration of follow-up [1]. Most importantly they examined different serum or urinary markers of bone turnover which are not well documented and in variable timeframes.

Currently, the measurements of serum levels of C-telopeptide fragments of collagen type I a1 chains (*β*-CTx) and N-terminal propeptide of type I procollagen (PINP) are recommended as markers of bone resorption and bone formation markers, respectively, correlated with corresponding histomorphometric parameters of bone formation and resorption [7]. However this recommendation is applicable to assess progression of osteoporosis, fracture risk, and treatment response.

Serum *β*-CTx is a bone resorption marker of choice in view that the assay is well characterized, measures an 8-amino-acid peptide, has been evaluated in many studies, and is widely available as an automated immunoassay or manual ELISA, and the biological and analytical variability is well documented [7]. Serum PINP is the reference standard for bone formation, because most PINP probably is produced during bone formation, the assay is widely available as an automated immunoassay or manual radioimmunoassay, and the biological and analytical variability is well known [7]. These two markers have been studied extensively in patients with osteoporosis but were only occasionally evaluated in critically ill patients and especially *β*-CTx in only two studies and PINP in another [4, 8, 9]. Therefore more data are needed to establish their validity in this heterogeneous group of patients.

The aim of the study was to measure the changes of the levels of two biochemical markers of bone metabolism PINP and *β*-CTx along with total 25-hydroxyvitamin D (25-OH vitamin D), and intact parathormone (iPTH) serum levels in critically ill patients during their ICU stay until discharge.

## 2. Patients and Methods

### 2.1. Study Design

In this prospective observational study we enrolled adult patients admitted to the ICU requiring mechanical ventilation expected to have at least 10 days of ICU stay and until discharge. Exclusion criteria included pediatric patients, pregnancy, renal, metabolic, and liver disease, metabolic and neurological disease, known osteoporosis, and prior medications affecting bone metabolism.

We prospectively collected demographics, admission diagnosis, common laboratory data routinely monitored for critically ill patients, APACHE II score, SOFA score, length of stay (LOS), drug administered including that affecting bone turnover such as low molecular weight heparin (LMWH), phenytoin, low dose steroids for septic shock (50 mg × 4 i.v.), and transfusions.

Fourteen healthy volunteers matched for age and sex (7 males and 7 females) of mean age (yrs) 65.2 ± 6.4 (median; 59, range 52–75 years) served as controls for *β*-CTx, PINP, iPTH, and total 25-hydroxyvitamin D serum levels for comparisons. These were historical samples randomly selected from a pool of data kept in the biochemistry department by selecting from a list one individual every five individuals until reaching 7 individuals.

### 2.2. Biochemical Measurements

Peripheral blood was collected at each time-point, between 08.00 and 10.00 A.M placed in tubes containing EDTA, centrifuged, and then stored in polypropylene tubes at −20°C until measurements. Time-points were baseline (within 24 h of ICU admission) and on a weekly basis until ICU discharge or death. Blood collection was performed at least 5 days far from death. Serum levels of iPTH, 25-OH vitamin D, *β*-CTx, and PINP were determined with an electrochemiluminescence immunoassay (ECLIA) on Cobas e411 (Roche, Manheim, Germany). The sensitivity of this assay for iPTH is 1.2 pg/mL, and the intra- and interassay CVs are 4 and 4.3%, respectively. The sensitivity of this assay for 25-hydroxyvitamin D is <5.0 ng/mL, and the intra- and interassay CVs are 4.4 and 4.7%, respectively. The total precision (CVt) of these assays for *β*-CTx and PINP is <3.5%, <1.8% and <4.5%, respectively.

### 2.3. Ethics

The study protocol was approved by the Scientific and Ethics Committees of the Hospital and waived the need for informed consent due to the nature of the study design. As patient care in the ICUs includes routine blood sampling for laboratory tests, no written informed consent was required from the patients.

### 2.4. Statistical Analysis

Qualitative characteristics are presented as numbers and percentages. Numerical data are expressed in mean ± SEM or SD or percentages. BTMs values on figures and the table are presented as median (±IQR).

Comparisons were performed using nonparametric test Kruskal-Wallis test with Dunn's posttest analysis suitable for multiple comparisons (between all pairs). Statistical significance was considered at the level of* p* < 0.05.

## 3. Results

### 3.1. Patient Demographics

We enrolled 28 patients (16 males) fulfilling the inclusion criteria. Patient demographics, medications, and laboratory measurements on admission are shown in Table 1. All patients had a pre-ICU stay in hospital wards or emergency department ranging from 1 to 3 days. All patients received enteral nutrition and supplemental micronutrients (Addamel™ N 10 mL, Fresenius Kabi USA, LLC) and vitamins (Cernevit™, Baxter Healthcare Ltd., A, B complex, C, D, E, and folic acid) in enriched intravenous fluids as medically indicated by physicians in charge. None of the patients experienced altered renal function tests (Table 1) that could influence bone turnover markers clearance from the circulation and thus their serum levels.

### 3.2. Markers of Bone Turnover 

#### 3.2.1. Control Group Values

BTMs for controls are shown in Table 2 together with consecutive BTM values for the patients.

#### 3.2.2. Bone Resorption

**Figure 1:**
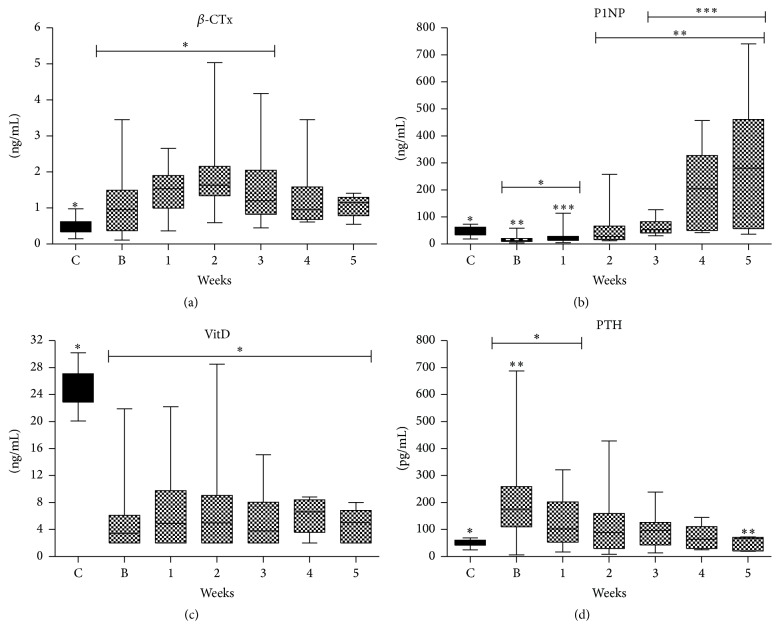
BTMs course during ICU stay in whole population. (a) CTx; (b) PINP; (c) Vitamin D; (d) Parathormone. Values are presented as media (±IQR). C = controls; B = baseline; numbers: weeks. Symbols *∗*, *∗∗*, and *∗∗∗* indicate statistically significant difference (*p* < 0.05).

Serum *β*-CTX levels were normal (Table 2, Figure 1(a)) in 55.2% of the patients on admission (baseline). Serum levels were increased in 82.1%, 84.2%, 60%, 50%, and 57.1% of the patients on 1st, 2nd, 3rd, 4th, and 5th week, respectively. On the 2nd week (peak levels) *β*-CTX serum levels were 2-fold times greater compared to controls. There was a statistically significant difference in *β*-CTX levels between controls and baseline, 1st, 2nd, and 3rd week (Figure 1(a)).

#### 3.2.3. Bone Formation

Serum PINP (Table 2, Figure 1(b)) were normal in 44.8% of the patients on admission (baseline) and below normal in the rest of the patients. On the 1st week serum levels were normal in 50%, were below normal in 39.3%, and above normal in 10.7% of the patients. On the 2nd week serum levels were normal in 63.2%, were below normal in 10.5%, and above normal in 26.3% of the patients. On the 3rd, 4th, and 5th week serum levels were normal in 60%, 37.5%, and 28.6% and above normal in 40%, 62.5%, and 71.4% of the patients, respectively. There was a statistically significant difference between controls and baseline and 1st week; between baseline and 2nd, 3rd, 4th, and 5th week; and between 1st and 3rd, 4th, and 5th week. Serum levels were increased by 16-fold on 4th and 22-fold on 5th week compared to baseline values. On 4th week and afterwards none of the patients had below normal levels.

#### 3.2.4. Parathyroid Hormone and 25-Hydroxyvitamin D

All patients had decreased 25-OH vitamin D serum levels on ICU admission compared to controls and were not restored to normal levels during the whole ICU stay (Figure 1(c)). Controls had significantly higher levels compared to the patients at all time-points. In parallel hypocalcaemia was observed at baseline (Table 1).

At baseline, 86.2% of the patients had increased serum iPTH levels which were 4-fold times greater than controls. On 2nd, 3rd, and 4th week 57.9%, 50%, and 28.5% of the patients still had increased iPTH values (Figure 1(d)). There was a statistically significant difference between controls and baseline and 1st week and between baseline and 5th week serum levels. We did not find any correlation between severity of illness (APACHE II) and iPTH or 25-OH vitamin D levels. Also, no correlation was found among iPTH and CTX values on admission, nor among iPTH and illness severity, ICU LOS, and SOFA scores.

#### 3.2.5. Measurements according to Survival

**Figure 2:**
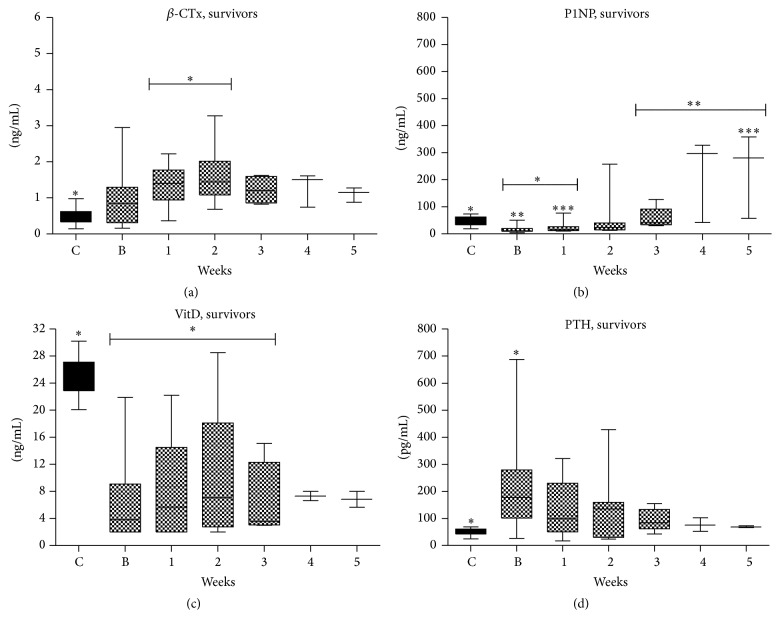
BTMs course during ICU stay in survivors. (a) CTx; (b) PINP; (c) Vitamin D; (d) Parathormone. Values are presented as media (±IQR). C = controls; B = baseline; numbers: weeks. Symbols *∗*, *∗∗*, and *∗∗∗* indicate statistically significant difference (*p* < 0.05).

**Figure 3:**
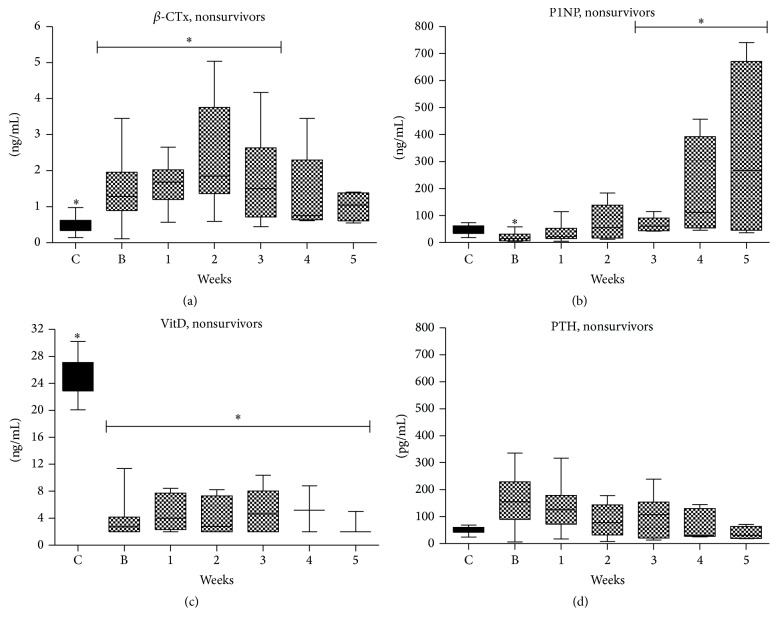
BTMs course during ICU stay in nonsurvivors. (a) CTx; (b) PINP; (c) Vitamin D; (d) Parathormone. Values are presented as media (±IQR). C = controls; B = baseline; numbers: weeks. Symbols *∗*, *∗∗*, and *∗∗∗* indicate statistically significant difference (*p* < 0.05).

We also performed analysis according to outcomes (Table 3 and Figures 2 and 3). Survivors had significantly shorter duration of mechanical ventilation (MV) (*p* = 0.039) and duration of ICU stay (*p* = 0.01) from nonsurvivors. Both survivors and especially nonsurvivors 25-OΗ vitamin D serum levels were significantly lower compared to normal throughout the whole ICU stay. iPTH serum levels were in both groups higher on admission tending to normalization on 4th to 5th week of ICU stay. *β*-CTx serum levels were higher compared to controls on admission and increased until the 2nd week of ICU stay in both survivors and nonsurvivors. On 3rd week and thereafter *β*-CTx serum levels decreased in both groups but were still higher compared to controls. PINP serum levels were lower compared to controls in both groups on admission but were increased overcoming control levels on 4th and 5th week of ICU stay in both groups.

## 4. Discussion

The major findings of this study are (1) critically ill patients had very low serum levels of vitamin D during ICU stay, whereas increased iPTH levels on admission were normalized in the majority of patients by the 4th week. (2) *β*-CTx serum levels were increased during ICU stay with a peak on 2nd week. (3) PINP serum levels were lower compared to controls on admission but increased significantly on the 4th to 5th week (5-fold) of follow-up.

Overall we observed a change of BTMs from increased bone resorption especially through the first 2 weeks of ICU stay, to increased bone formation after the 3rd week. Our findings suggest a biphasic model of BTMs changes that is of extensive initial bone resorption pattern which is then partially counterbalanced by bone formation. Our findings are also indicative of altered bone turnover in a selected population of critically ill patients which is consistent with current literature showing an association between the duration of critical illness (>5 days) and bone resorption [1, 10]. This BTMs pattern is consistent with previous studies that reported increased bone resorption markers [4, 8–14], increased serum osteoclast precursors [15], increased bone formation, and decreased osteocalcin during critical illness compared with controls [4, 11, 16]. These studies consistently described changes: an increased osteoclastic bone resorption (increased urinary DpD and PyD, serum B-CTX/ICTP), an increase in immature osteoblast number and activity (serum P1CP and PINP), and a reduced activity of mature osteoblasts (serum OC and ALP) [4, 8, 10–17].

Our biphasic model of BTMs changes cannot be generalized in every ICU patient and may be partially explained due to longer follow-up period (median 21 days) compared to previous studies in which the follow-up period was up to 10 days [4, 8, 10–17]. We also had baseline and weekly BTMs measurements and not single measurement on admission or discharge as previous studies [1].

We measured serum PINP and *β*-CTX levels as the most promising BTMs by the Joint International Bone Markers Standards Working Group [7] which however were occasionally evaluated and especially *β*-CTX in two studies and PINP in one study. Consistently to our results *β*-CTX was increased by 3- to 6-fold compared to controls or reference values in two studies [4, 8]. Also, PINP was increased by 1.7-fold on ICU discharge [4]. However these studies monitored patients for up to 10 days and measured BMT only once without evaluating calcium, iPTH, or vitamin D.

A very recent study found increased CTx and decreased serum PINP levels during ICU stay which is in accordance with our findings [9]. In addition to this study we found a commensurate response in bone formation markers during critical illness, that is, after the 3rd week of ICU stay as stated above. However, we have significant methodological differences compared to this study because we enrolled both survivors and not nonsurvivors, patients with longer duration of MV and ICU LOS, and multiple time-points of BTMs measurements during ICU stay.

In accordance with our study patients had during their ICU stay insufficient vitamin D serum levels, increased iPTH serum levels, and hypocalcaemia [9]. It was also suggested that vitamin D deficiency in critically ill patients with resultant secondary hyperparathyroidism along with prolonged immobilization and other metabolic abnormalities may increase the risk of excessive bone resorption [1, 17]. However, correction of vitamin D deficiency in critically ill patients did not normalize bone resorption markers, suggesting vitamin D deficiency is not the only mechanism for accelerated bone turnover [4, 12]. Hypovitaminosis D is common and multifactorial in critically ill patients but several pathophysiological and iatrogenic interventions and particularly during acute hemodilution have impact on 25-OH-D assay accuracy and results, making the assessment of vitamin D status during critical care hazardous. Also, the best option to measure 25-OH-D blood concentration has not been definitely defined [18].

### 4.1. Limitations of the Study

An important limitation of our study is that we do not report changes in bone mineral density (BMD) during or following critical illness. However, measurements of BTMs on ICU discharge may be a useful tool to identify patients requiring BMD measurement after ICU. Also, the relatively small number of patients, the short duration of follow-up, the absence of post-ICU follow-up, and a lack of premorbid assessment of BTMs or skeletal health may be disadvantages of the study. The short duration of follow-up decreases the ability to establish a causal relationship between critical illness and bone turnover [1].

Another important limitation of the study is the lack of analysis of the effect of possible confounding variables on the association between critical illness and altered bone turnover. The effects of other known causes of osteoporosis and variables, known to affect the metabolism of BTMs (including sex, menopausal status, renal failure, liver disease, diabetes, thyroid disease, inflammation, and medications and particularly glucocorticoids), were not consistently addressed. Also, the use of low dose hydrocortisone as part of sepsis management in most of the patients (≈61%) could affect markers levels. Also, infections like pneumonia may affect PINP levels in the circulation [19]. Finally the study was not designed to identify differences among survivors and nonsurvivors and thus further analysis could not be performed.

## 5. Conclusions

In this prospective observational study we found that critically ill patients receiving MV had a pattern of hypovitaminosis D, increased iPTH, hypocalcaemia, and a BTM compatible with altered bone metabolism and accelerated bone turnover consistent with increased bone loss and only partial commensurate response regarding bone formation. This model of biphasic BTMs changes in critically ill patients during ICU stay is described for first time. Our findings suggest that further studies are needed to confirm our data incorporating BMD changes during and after critical illness, identifying risk factors and the extent of critical illness related to bone turnover changes. Finally, more studies are needed to design appropriate preventive measures including physical therapy and therapeutic strategies.

## Figures and Tables

**Table 1:** Demographics, admission diagnosis, and characteristics of the patients (*n* = 28).

Variable	Values
Age, (range) yrs	67,4 ± 2,1 (45–88)
Male, *n*, %	16, (57,1)
Admission diagnosis, *n*	
Pulmonary Infection, RR	15
Pulmonary edema, CHF	5
Complicated surgery	4
SAH/ICH	2
Abdominal sepsis	1
Trauma, head injury	1
Type, *n*	
Medical	24
Surgical	4
APACHE II	22,2 ± 0,9
Predicted death rate, %	43,1
SOFA	10,1 ± 0,6
Outcome (mortality %)	10/28 (35,7)
MV duration, days	24,5 ± 5,1
ICU LOS (median), days	31,6 ± 5,7 (21)
Fever, >38.2°C	12/28
Low-dose steroids, *n*	17/28
Shock, nor-adrenaline, *n*	27/28
LMWH, *n*	17/28
Fondaparinux (Arixtra), *n*	9/28
Transfusions, *n*, (mean)	18/28 (2,9 ± 0,7 units)
Phenytoin (Epanutin), *n*	3/28
Albumin, g/dL	3,1 ± 0,1 (3,7–5,2)
Corrected calcium, mg/dL	8,2 ± 0,3 (8,5–10,5)
Phosphorus, mg/dL	3,4 ± 0,2 (2,5–4,8)
Alkaline phosphatase, U/L	120,0 ± 1,4 (42–141)
Urea, mg/dL	53,9 ± 5,3 (10–50)
AST (SGOT), IU/dL	44,4 ± 9,8 (9–36)
ALT (SGPT), IU/dL	68,9 ± 28,9 (20–28)
WBC, /mm^3^	11,087 ± 1,415
Creatinine, mg/dL	1,1 ± 0.7 (0,6–1,4)
CRP, mg/dL	108,4 ± 20,1 (<3,16)
Food supplementation, *n*	
Enteral	28/28
Parenteral	2
Both	2/28
Micronutrients	28/28
Vitamins	28/28

In parenthesis data represents range,* n*, percentages, or normal values. RR: respiratory failure, ICH: intracerebral hemorrhage, SAH: subarachnoid hemorrhage, CHF: congestive heart failure, LMWH: low molecular weight heparin, CRP: c-reactive protein, ICU: intensive care medicine, WBC: white blood cell, APACHE II: Acute Physiology And Chronic Health Evaluation, SOFA: Sequential Organ Failure Assessment, AST: Aspartate Aminotransferase, and ALT: Alanine Aminotransferase.

**Table 2:** Values for BTMs obtained from controls versus patients over time (weeks) of ICU stay.

Parameter	Controls (*n* = 14)	Baseline (*n* = 28)	1st (*n *= 28)	2nd (*n* = 19)	3rd (*n* = 11)	4th (*n* = 8)	5th (*n* = 7)
*β*-CTX, ng/mL	0.47 (0.34–0.63)	0,95 (0,37–1,49)	1,54 (0,99–1,90)	1,64 (1,34–2,15)	1,2 (0,83–2,05)	0,96 (0,68–1,59)	1.15 (0,79–1,30)
P1NP, ng/mL	45.5 (33.7–62.1)	12,75 (9,53–20,56)	20,6 (13,7–28,5)	27,4 (16,1–66,5)	52,8 (41,1–82,8)	204,3 (50,1–327,8)	281,1 (56,8–460,7)
iPTH, pg/mL	49.5 (42.3–60.4)	175,4 (110,5–258,9)	102,5 (53,2–202,5)	88,4 (29,8–159)	96,5 (42,8–126,5)	63,7 (29,8–110,9)	66,1 (20,2–71,3)
25-OH VitD^*∗*^	24.1 (22.9–27.1)	3,4 (2,0–6,1)	4,9 (2,0–9,8)	4,9 (2,0–9,1)	3,8 (2,0–8,1)	6,6 (3,6–8,4)	5,1 (2,0–6,8)

^*∗*^ng/mL values are expressed as median (±IQR).

**Table 3:** Demographics, admission diagnosis, and characteristics of the patients according to survival status (*n *= 28).

Variable	Survivors (*n* = 18)	Nonsurvivors (*n* = 10)	*p*
Age, (range) yrs	65.0 ± 2.5	71.6 ± 3.2	0.88
Males, *n*, %	10/18, 55.5%	6/10, 60.0%	1.0
Admission diagnosis, *n*			
Pulmonary Infection, RR	10	5	
Pulmonary edema, CHF	3	2	
Complicated surgery	2	2	
SAH/ICH	1	1	
Abdominal sepsis	1		
Trauma, head injury	1		
Type, *n*			
Medical	16	8	
Surgical	2	2	
APACHE II	21.9 ± 1.2	22.7 ± 1.5	0.58
SAPS II	62.9 ± 2.5	60.1 ± 3.5	0.83
SOFA	9.9 ± 0.9	10.5 ± 0.9	0.65
MV duration, days	14.3 ± 2.8	42.8 ± 11.7	0.039
ICU LOS (median), d	18.6 ± 2.9 (13)	55.0 ± 12.1 (51.5)	0.01
Fever, >38.2°C	8/18, 44,4%	4/10, 40%	1.0
Corrected calcium, mg/dL	7.9 ± 0.2	8.5 ± 0.6	0.65
Phosphorus, mg/dL	3.2 ± 0.3	3.8 ± 0.3	0.13
Alkaline phosphatase, mg/dL	117.7 ± 19.5	124.0 ± 17.1	0.18
Urea, mg/dL	54.5 ± 7.1	52.8 ± 8.3	0.96
AST (SGOT), IU/dL	51.4 ± 14.9	31.8 ± 4.6	1.0
ALT (SGPT), IU/dL	90.1 ± 44.4	31.1 ± 5.9	0.98
WBC, /mm^3^	10,964 ± 1,997	11,309 ± 1,804	0.79
Creatinine, mg/dL	1.1 ± 0.8	1.2 ± 0.2	0.51
Albumin, g/dL	3.0 ± 0.1	3.3 ± 0.2	0.38
CRP, mg/dL	112.1 ± 24.2	97.8 ± 38.9	0.75
i-CTx (ng/mL)	0,96 ± 0,19	1,49 ± 0,32	0.098
P1NP (ng/mL)	15,46 ± 2,44	19,85 ± 5,34	0.773
i-PTH (pg/mL)	215,9 ± 39,68	159,6 ± 32,68	0.533
25-OH Vit. D (ng/mL)	6,45 ± 1,49	3,80 ± 0,94	0.433
Low-dose steroids, *n*	12/18	4/10	
Shock, nor-adrenaline, *n*	17/18	10/10	
LMWH, *n*	14/18	5/10	
Fondaparinux (Arixtra), *n*	4/18	5/10	
Transfusions, *n*, (mean)	10/18	8/10	
Phenytoin (Epanutin), *n*	2/18	1/10	
Food supplementation, *n*	18/18	10/10	
Enteral	18/18	10/10	
Parenteral	1/18	1/10	
Both	1/18	1/10	
Micronutrients	18/18	10/10	
Vitamins	18/18	10/10	

RR: respiratory failure, ICH: intracerebral hemorrhage, SAH: subarachnoid hemorrhage, CHF: congestive heart failure, LMWH: low molecular weight heparin, CRP: c-reactive protein, ICU: intensive care medicine, WBC: white blood cell, APACHE II: Acute Physiology And Chronic Health Evaluation, SOFA: Sequential Organ Failure Assessment, AST: Aspartate Aminotransferase, and ALT: Alanine Aminotransferase.

## Abbreviations

APACHE II score: Acute Physiologic Assessment and Chronic Health Evaluation
BMD: Bone mineral density
BTMs: Bone turnover markers
*β*-CTx: C-telopeptide fragments of collagen type I a1 chains
ICU: Intensive care unit
iPTH: Intact parathormone
LOS: Length of stay
LMWH: Low molecular weight heparin
MV: Mechanical ventilation
PINP: N-terminal propeptide of type I procollagen
25-OH vitamin D: total 25-hydroxyvitamin D
SOFA score: Sepsis-Related Organ Failure Assessment.
